# Talaromycosis-associated immune reconstitution inflammatory syndrome exhibits immune dysregulation in patients with acquired immunodeficiency syndrome

**DOI:** 10.1371/journal.pntd.0014695

**Published:** 2026-09-01

**Authors:** Bei Zhao, Qinzhi Zhang, Pengle Guo, Yingyin Yang, Feilong Xu, Xuemei Ling, Kaiyin He, Xiejie Chen, Xiaoping Tang, Huihua Zhang, Linghua Li

**Affiliations:** 1 Infectious Disease Center, Guangzhou Eighth People’s Hospital, Guangzhou Medical University, Guangzhou, China; 2 Guangzhou Medical Research Institute of Infectious Diseases, Guangzhou Eighth People’s Hospital, Guangzhou Medical University, Guangzhou, China; 3 Infectious Diseases Department, Foshan First People’s Hospital, Foshan, China; Gulu University, UGANDA

## Abstract

**Background:**

Talaromycosis (TSM) is a severe opportunistic fungal infection that mostly infects patients with acquired immunodeficiency syndrome (AIDS). After antiretroviral therapy (ART), AIDS patients with TSM may develop immune reconstitution inflammatory syndrome (IRIS), leading to the paradoxical worsening of clinical symptoms. However, the immunological characteristics of TSM-related IRIS (TSM-IRIS) in AIDS patients remain poorly understood.

**Methodology/Principal Findings:**

We conducted a longitudinal cohort study of AIDS patients with TSM and analyzed the immunological features using flow cytometry, transcriptome, and multiplex cytokine assays at admission, ART initiation, and at 2, 4 weeks after ART initiation. Patients with TSM-IRIS displayed significantly higher baseline CD3 ⁺ T cells at admission and lower frequencies of PD-1 ⁺ CD4 ⁺ T cells at ART initiation than those without IRIS (non-IRIS). After ART initiation, TSM-IRIS was associated with dynamic transcriptomic changes involving inflammatory and immune-related pathway enrichment, along with sustained elevations of interleukin (IL)-17, IL-21, C-X-C motif chemokine ligand (CXCL) 5, and CXCL12. Transcriptomic analysis revealed the dynamic enrichment of pro-inflammatory pathways prior to and during the early phase of ART.

**Conclusion/Significance:**

AIDS patients with TSM-IRIS exhibited an immune profile associated with enhanced T cell activation, characterized by accelerated immune reconstitution accompanied by heightened inflammatory activity during the early phase of ART, may potentially acting as the immune markers for prediction or diagnosis of TSM-IRIS in AIDS patients.

## Author summary

For patients with acquired immunodeficiency syndrome (AIDS), initiating antiretroviral therapy (ART) typically restores immune function and improves clinical outcomes. However, following ART initiation, restored immunity to specific infectious or non-infectious antigens may result in paradoxical clinical worsening or new disease. In this study, we monitored and compared immunological features in AIDS patients with talaromycosis (TSM) who developed immune reconstitution inflammatory syndrome (IRIS) with those without IRIS during ART treatment. We found that patients with TSM-IRIS exhibited an earlier rise in CD3^+^ T cells at admission and lower frequencies of PD-1^+^ CD4^+^ T cells at ART initiation than patients without IRIS. Overall, our study described the accelerated immune reconstitution accompanied by heightened inflammatory activity in TSM-IRIS, a phenomenon with limited prior evidence.

## Introduction

Talaromycosis (TSM) is an often-overlooked opportunistic infection caused by *Talaromyces marneffei* (*T. marneffei*) [1,2]. However, TSM has emerged as a leading cause of mortality, accounting for approximately 33% of deaths among patients with advanced acquired immunodeficiency syndrome (AIDS), particularly in Thailand, Vietnam, and Southern China [3,4]. Antiretroviral therapy (ART) can improve patients’ prognosis by restoring immune function [5]. However, in AIDS patients with TSM, immune recovery following ART may paradoxically trigger TSM-associated immune reconstitution inflammatory syndrome (IRIS) [6].

IRIS exhibits pathogen-specific immunological signatures, as shown in studies of tuberculosis- and cryptococcosis-associated IRIS, where excessive immune activation and exaggerated inflammatory responses have been well described [7,8]. In contrast, the immunological features of TSM-associated IRIS (TSM-IRIS) remain poorly characterized, with existing evidence primarily limited to case reports [9,10]. Our previous study demonstrated that TSM-IRIS exhibited higher CD4^+^ T cells, accelerated immune recovery and prolonged hospitalization [11]. However, the immunological features of TSM-IRIS except CD4^+^ T cells remain unclear. To address these knowledge gaps, we conducted a longitudinal cohort study to systematically characterize the immunological features of TSM-IRIS in AIDS patients during ART treatment.

## Methods

### Ethics statement

This study was approved by the Ethics Committee of Guangzhou Eighth People’s Hospital, Guangzhou Medical University (Approval No. 202034167). The participants were enrolled from a cohort registered at ClinicalTrials.gov (NCT04667026). Written informed consent was obtained from all participants.

### Study cohort and clinical specimens

This study enrolled hospitalized AIDS patients who were diagnosed with TSM at Guangzhou Eighth People’s Hospital, Guangzhou Medical University, between June 2021 and June 2024. The inclusion criteria were as follows: (1) age ≥ 18 years; (2) confirmed diagnosis of human immunodeficiency virus (HIV) infection; (3) confirmed TM infection based on positive blood and/or bone marrow and/or other body fluids culture; (4) ART-naïve or re-initiating ART after an interruption of >24 weeks; and (5) availability of complete sample sets, including plasma and peripheral blood mononuclear cell (PBMC) collected at admission (baseline), prior to ART initiation, 2 and 4 weeks after ART initiation. The exclusion criteria were as follows: (1) co-infection with *Mycobacterium tuberculosis* or *Cryptococcus* spp., (2) presence of malignant tumors, and (3) follow-up duration of less than 4 weeks after ART initiation. Other concomitant opportunistic infections were recorded at baseline but were not excluded, because these associated IRIS were relatively uncommon. Patients who developed IRIS during follow-up were classified into the TSM-IRIS group, whereas those without IRIS were categorized into the non-IRIS group.

TSM-IRIS was diagnosed using criteria described in our previous study refer to the standards in the EACS guideline 2020 [11]. Briefly, TSM-IRIS was defined based on two required criteria: (1) the exacerbation of clinical symptoms specifically attributed to TSM after ART initiation, and (2) exclusion of the possibility of treatment failure, drug-related adverse effects, and new infections. IRIS events were defined as those occurring within 3 months after ART initiation. Both criteria were required for diagnosis, and all suspected IRIS events were independently adjudicated by two experienced physicians. As all patients had confirmed *T. marneffei* infection before ART initiation, TSM-associated IRIS in this study referred specifically to paradoxical IRIS. Considering the relatively low incidence of TSM-IRIS, all eligible patients with IRIS with complete longitudinal sampling were included. Patients in the non-IRIS group were selected during the same study period. The two groups were compared in terms of age and sex distribution to minimize potential confounding factors.

### Sample collection

Peripheral blood was collected into ethylenediaminetetraacetic acid (EDTA) anticoagulated tubes and processed within 6 h of collection. Blood samples were centrifuged at 3,000 rpm for 6 min to separate plasma, which was aliquoted and stored at −80°C. The remaining cellular fraction was diluted with RPMI-1640 medium and subjected to density-gradient centrifugation using lymphocyte separation medium. The PBMC layer at the interface was carefully collected, washed with RPMI-1640 medium supplemented with 10% fetal bovine serum, and resuspended in freezing medium containing 90% fetal bovine serum and 10% dimethyl sulfoxide. PBMCs were gradually cooled to −80°C and then transferred to liquid nitrogen within 48 h for long-term storage.

### Flow cytometry

Cryopreserved PBMCs were rapidly thawed, washed, and then stained with the following fluorochrome-conjugated monoclonal antibodies: anti-human CD8 Brilliant Violet 570, anti-human CD45RA FITC, anti-human CD28 PE/Dazzle 594, anti-human CD19 PerCP-Cy5.5, anti-human CCR7 (CD197) PE-Cy7, anti-human CD56 (neural cell adhesion molecule, NCAM) Alexa Fluor 700, anti-human CD14 PE, anti-human CD4 Brilliant Violet 510, anti-human CD11b Brilliant Violet 650, anti-human PD-1 (CD279) Brilliant Violet 711, and anti-human HLA-DR (human leukocyte antigen-DR isotype) Brilliant Violet 605 (all from BioLegend, San Diego, CA, USA) and anti-human CD3 Alexa Fluor 532, anti-human CD16 eFluor 450 (Thermo Fisher Scientific, Waltham, MA, USA depending on reagent). A fixable viability dye (Zombie NIR; BioLegend) was used to exclude dead cells. Samples were acquired on a Cytek full-spectrum flow cytometer and analyzed using FlowJo software (BD Biosciences, Franklin Lakes, NJ, USA).

### Sorting strategy

PBMCs were isolated using density gradient centrifugation and analyzed by flow cytometry. Live singlet lymphocytes were gated and separated into CD3^+^ and CD3^-^ populations. Cells were sorted from gated live singlet CD3^+^ lymphocytes based on the following expression patterns: CD4^+^ PD-1^+^, CD4^+^ CD28^+^, CD8^+^ PD-1^+^, CD8^+^ CD28^+^, Naïve CD8 (CD8^+^ CD45RA^+^CCR7^+^), central memory CD8 (CD8^+^ CD45RA^-^CCR7^+^), effector memory CD8 (CD8^+^CD45RA^-^CCR7^-^), and T effector CD8 (CD8^+^CD45RA^+^CCR7^-^). Within CD3^-^ cells^-^, CD11b^+^ monocytes were subdivided into classical (CD14^++^CD16^-^), non-classical (CD14^+^CD16^++^), and intermediate (CD14^++^CD16^+^) subsets, while natural killer (NK) cells (CD16^+^CD56^+^) and B cells (CD19^+^) were defined from the CD11b^-^ fraction (S1 Fig).

### RNA extraction and sequencing

Strand-specific RNA-seq libraries with unique indexes were prepared using the VAHTS Universal V8 RNA-seq Library Prep Kit for MGI (Vazyme, Nanjing, China; Cat. No. NRM605), according to the manufacturer’s instructions. Briefly, the total RNA samples were subjected to fragmentation and cDNA synthesis, followed by adaptor ligation in a thermal cycler. The DNA libraries were subsequently purified using VAHTS magnetic beads (Vazyme, Nanjing, China; Cat. No. NRM605) according to the manufacturer’s protocol, which included bead activation, nucleic acid binding, ethanol washing, and final elution in nuclease-free water.

### Bioinformatic analysis

RNA quality was assessed using an Agilent 2100 bioanalyzer (Agilent, Santa Clara, CA, USA). High-throughput sequencing was performed using DNA nanoballs (DNB) based on an MGI-2000 platform. After the quality assessment, raw gene-level count data were generated using featureCounts and HTSeq. Differential expression and functional enrichment analyses were performed using DESeq2 and clusterProfiler, and heatmap plots were generated to visualize the enriched terms. Volcano plots of differentially expressed genes (DEGs) were generated using the ggplot2 package. DEGs were functionally clustered based on Kyoto Encyclopedia of Genes and Genomes database annotations, with enrichment tested using the R phyper function and adjusted for false discovery rate. In addition, gene set enrichment analysis was conducted based on Hallmark gene sets from the Molecular Signatures Database to further evaluate biological pathways enriched in the target gene set.

### Determination of cytokine in plasma

Plasma cytokine and biomarker concentrations were measured using the U-PLEX Biomarker Group 1 (human) Multiplex Assay (Meso Scale Discovery, USA) following the manufacturer’s instructions. The measured analytes included IL-17E, IL-17F, thymic stromal lymphopoietin (TSLP), CXCL5, IL-23, IL-31, IL-9, IL-17A/F, CXCL12, CCL11, CXCL1, CCL26, IL-21, IFN-γ1 and CCL24. Briefly, biotinylated capture antibodies were coupled to unique linkers and assembled into a coating solution, which was applied to U-PLEX 96-well plates. After blocking and washing, the plasma samples were diluted. Following incubation, the plates were washed and incubated with a cocktail of detection antibodies and then developed with MSD GOLD Read Buffer B. Signals were acquired on an MSD instrument and cytokine concentrations were calculated.

### Statistical analysis

Quantitative data collected at different time points were compared using One-Way ANOVA following multiple comparisons. Quantitative variables were compared between the two groups at the same time point using Two-Way ANOVA following multiple comparisons. Categorical variables differences between groups were assessed using the chi-square (χ^2^) test or Fisher’s exact test. All statistical analyses were conducted using R (version 4.5.2) and graphs were generated using R and GraphPad Prism version 8.0 (GraphPad Software, San Diego, CA, USA). A *P* value < 0.05 was considered statistically significant.

## Results

### Characteristics of the study patients

Twenty-six AIDS patients with TSM were enrolled in this study. Among them, 11 (42%) developed IRIS and 15 not. All patients had disseminated *T. marneffei* infection, as evidenced by positive blood cultures (Table 1). On admission, no statistically significant differences in clinical variables were observed between the two groups, including CD4 ⁺ T cells, CD4 ⁺ /CD8 ⁺ ratios, viral loads, concomitant opportunistic infections and other clinical variables (Table 1 and S1 Table). Most patients received amphotericin B-based treatment, and the distribution of antifungal regimens did not differ significantly between the IRIS and non-IRIS groups. ART was initiated a median of 12 days after the start of antifungal therapy in both groups). In IRIS group, the median time from ART initiation to IRIS onset was 15 days (IQR, 8–18 days). Fever was the most common clinical manifestation, and all patients with TSM-IRIS received systemic corticosteroids after IRIS diagnosis.

**Table 1:** Baseline demographic and laboratory characteristics of the study participants.

	Total (*n* = 26)	TSM-IRIS (n = 11)	non-IRIS (n = 15)	*P* value
Male (%)	24 (92.3)	10 (90.9)	14 (93.3)	1.000
Age, years, IQR	35 (30–44)	36 (35–50)	30 (28–38)	0.057
Interval between antifungal therapy and ART initiation (days, IQR)	12 (8–16)	11 (7–15)	12 (9–15)	0.567
ART regimen, n (%)				
NRTIs+ INSTIs	25 (96.2)	11 (100)	14 (93.3)	1.000
NRTIs+ NNRTIs	1 (3.8)	0	1 (6.7)	
Infection site, n (%)				
Blood	26 (100.0)	11 (100.0)	15 (100.0)	NA
Bone marrow	22 (84.6)	10 (90.9)	12 (80.0)	0.614
Lymph node	3 (11.5)	2 (18.2)	1 (6.7)	0.556
Lung	1 (3.8)	1 (9.1)	0	0.423
Concomitant opportunistic infections, n (%)	14 (53.8%)	7 (63.6)	7 (46.7)	0.453
*Nontuberculous mycobacteria*	4 (15.4)	2 (18.2)	2 (13.3)	1.000
*Cytomegalovirus*	5 (19.2)	2 (18.2)	3 (20.0)	1.000
*Pneumocystis jirovecii*	3 (11.5)	2 (18.2)	1 (6.7)	0.774
*Candida*	2 (7.7)	1 (9.1)	1 (6.7)	1.000
CD4 ^+^ T cells, cells/μL, IQR	9 (5–18)	10 (6–23)	7 (5–18)	0.581
CD4 ⁺ /CD8 ⁺ ratio	0.03 (0.03–0.07)	0.05 (0.04–0.09)	0.04 (0.02–0.07)	0.330
HIV-1 RNA, log_10_ copies/mL, IQR	5.80 (5.39–6.24)	5.83 (5.53–6.23)	5.75 (5.26–6.15)	0.477
CRP, mg/L, IQR	64.83 (37.15–101.08)	51.99 (22.4–93.39)	67.51 (50.16–107.21)	0.371
WBC, 10^9^/L, IQR	4.22 (2.83–6.5)	3.06 (2.62–5.1)	4.97 (3.48–8.13)	0.069
Mono, 10^9^/L, IQR	0.2 (0.09–0.3)	0.21 (0.07–0.31)	0.18 (0.11–0.29)	0.716
HGB, g/L, IQR	94.5 (82.75–107.25)	98 (86.5–109)	89 (80–102.5)	0.436
PLT, 10^9^/L, IQR	76.5 (46.5–198.5)	144 (45–191.5)	70 (48–193)	0.917
PCT, ng/mL, IQR	0.88 (0.37–2.14)	0.49 (0.24–1.54)	0.93 (0.6–2.57)	0.298
ALB, g/L, IQR	28 (23.78–31.48)	28.6 (23.9–30.75)	27.4 (24.05–32.25)	0.604
ALT, U/L, IQR	34.35 (25.03–62.23)	33.2 (27.7–69.35)	37.2 (22.75–58.25)	0.736
AST, U/L, IQR	71.05 (41.48–145.3)	67.8 (39.75–139.75)	74.3 (48.55–143.9)	0.979
Antifungal treatment, n (%)				
Amphotericin B-based	25 (96.2)	10 (90.9)	15 (100.0)	0.423
Voriconazole	1 (3.8)	1 (9.1)	0	0.423

Abbreviations: ART = antiretroviral therapy; NRTIs = nucleos(t)ide reverse transcriptase inhibitors; INSTIs = integrase strand transfer inhibitors; NNRTIs = non-nucleoside reverse transcriptase inhibitors; HIV = human immunodeficiency virus; RNA = ribonucleic acid; CRP = C-reactive protein; WBC = white blood cell count; HGB, hemoglobin; PLT = platelet count; PCT = procalcitonin; ALB = albumin; ALT = alanine aminotransferase; AST = aspartate aminotransferase.

### Longitudinal changes in immune cell subsets in IRIS and non-IRIS groups

**Fig 1:**
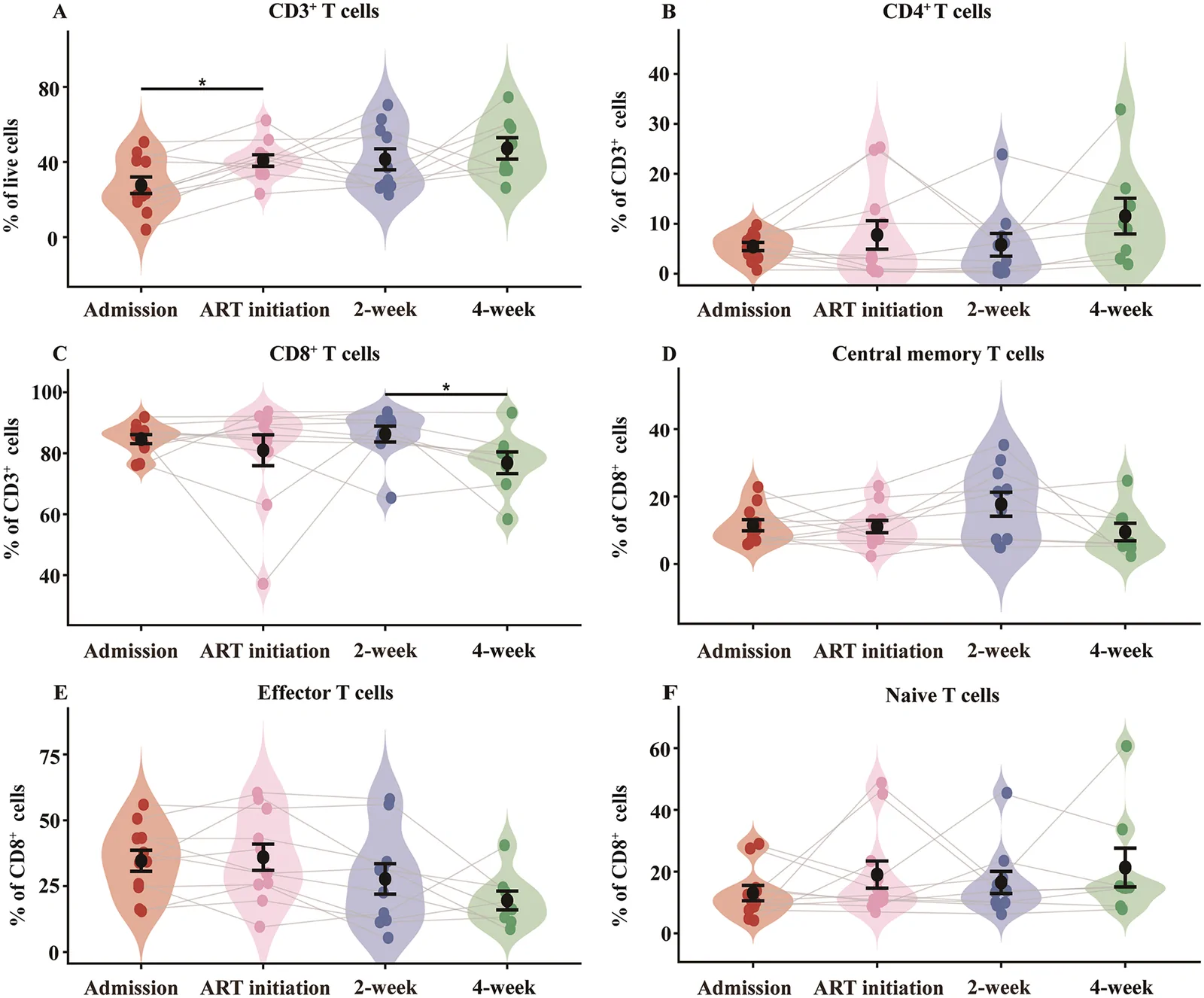
Comparison of total T cells and T cell subsets in the TSM-IRIS group at admission, ART initiation, and 2 and 4 weeks after ART. Violin plots depicts CD3 ⁺ T cells, CD4 ⁺ T cells, CD8 ⁺ T cells, and central memory T cells, effector memory T cells, and naïve T cell subsets. Black points and bars denotes mean ± SEM; gray lines connect paired samples. *P value < 0.05. ART = Antiretroviral therapy. Admission indicates the time of hospital admission. ART initiation indicates the pre-ART time point after initial antifungal treatment. “2-week” and “4-week” indicate 2 and 4 weeks after ART initiation, respectively.

Exploration of the spectrum of immune cell subsets showed that T cells in the TSM-IRIS group increased significantly at ART initiation compared with baseline (Fig 1A). However, CD4 ⁺ T cells did not change significantly across four time points (Fig 1B). CD8 ⁺ T cells at 4 weeks decreased markedly compared with those at 2 weeks after ART; however, no significant difference was observed at any other time point (Fig 1C). These findings suggest that the relative composition of the major CD4^+^ and CD8^+^ T cells subsets remained largely stable despite changes in the overall CD3^+^ T cells compartment. The frequency of PD-1 ⁺ CD8 ⁺ T cells declined from 2 weeks to 4 weeks, while PD-1 ⁺ CD4 ⁺ T cells remained relatively stable throughout the observation period (S2A-S2E Fig). No obvious differences were observed in NK, B cells, or intermediate monocytes across all time points (S2F-S2G and S2I Fig). Non-classical monocytes showed a downward tendency at 4 weeks compared with those at admission, while classical monocytes showed minimal fluctuations over time (S2H and S2J Fig). In the non-IRIS group, CD3⁺ T cells increased at ART initiation compared with those at admission, whereas CD4 ⁺ and CD8 ⁺ T cells showed no significant changes. Several immune cell subsets including CD28⁺ and PD-1 ⁺ T cell subsets, effector memory CD8 ⁺ T cells, NK cells, B cells, and monocyte subsets, exhibited statistically significant changes at specific time points (S3 and S4 Figs).

### Differences in immune cell subsets between IRIS and non-IRIS group

**Fig 2:**
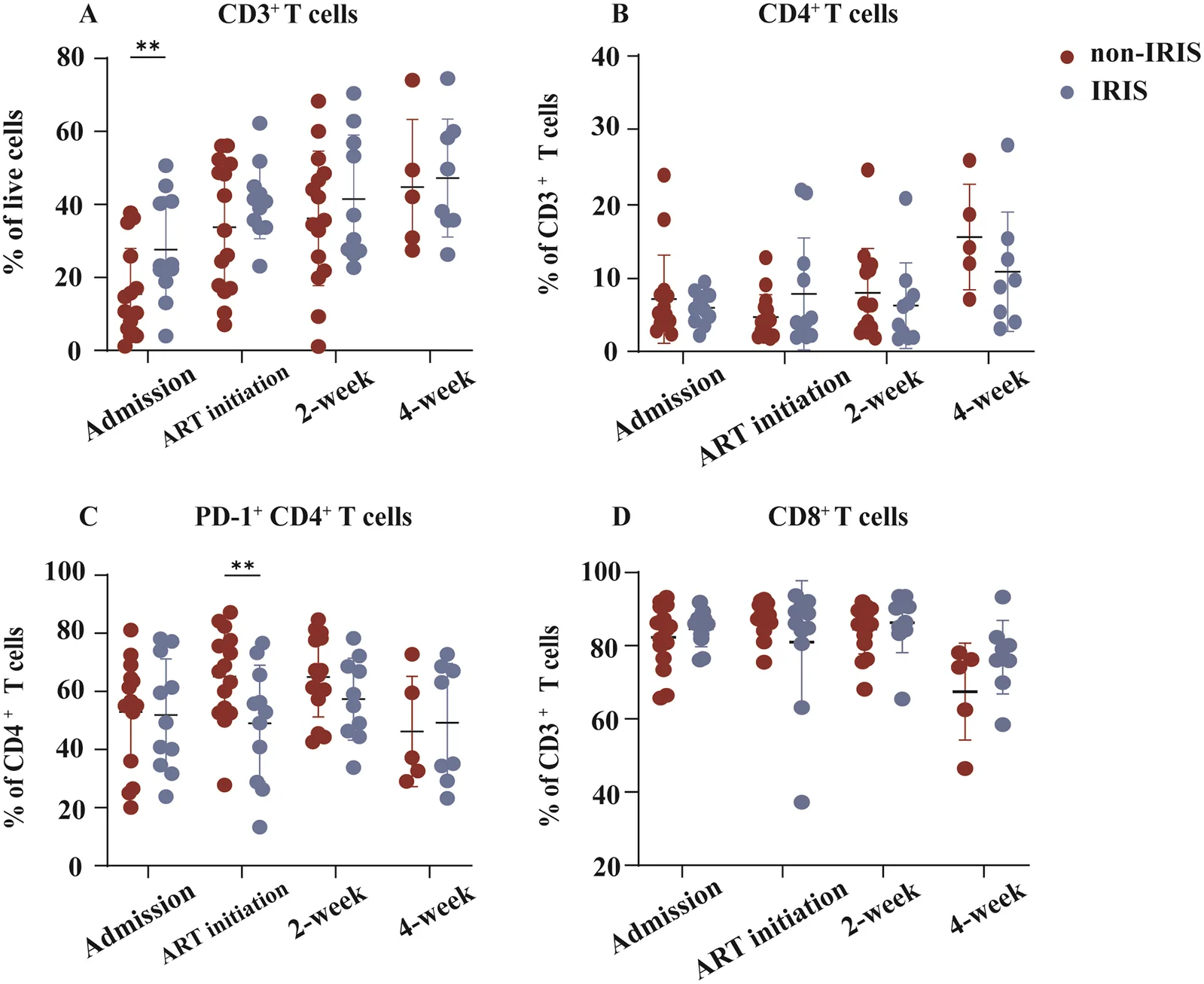
Comparison of total T cells and CD4^+^ T cell subsets between patients with TSM-IRIS and non-IRIS at different time points. Comparison of (A) total T cells, **(B)** CD4 ⁺ T cells; **(C)** PD-1 ⁺ CD4 ⁺ T cells, and **(D)** CD8 ⁺ T cells between the TSM-IRIS and non-IRIS groups. Bars denotes mean ± SEM. **P value < 0.01.

Comparison of immune cell subsets in TSM-IRIS and non-IRIS groups showed that CD3 ⁺ T cells were significantly higher in the TSM-IRIS group than those in the non-IRIS group at baseline (Fig 2A). No differences of CD4^+^ T cells between two groups across all time points (Fig 2B). At admission, the frequency of PD-1^+^ CD4^+^ T cells was similar between two groups. However, at ART initiation, IRIS group had a lower frequency of PD-1^+^ CD4^+^ T cells than non-IRIS group (Fig 2C), suggesting the divergence expression of PD-1 during the pre-ART antifungal treatment interval rather than being present at admission. No differences were observed in CD8 ⁺ T cells subsets (Fig 2D, S5B-S5G Fig). Moreover, the distributions of B cells, NK, and monocyte subsets were comparable between two groups (S5H-S5I Fig).

### Dynamic transcriptomic reprogramming and pro-inflammatory pathway activation in patients with TSM-IRIS

**Fig 3:**
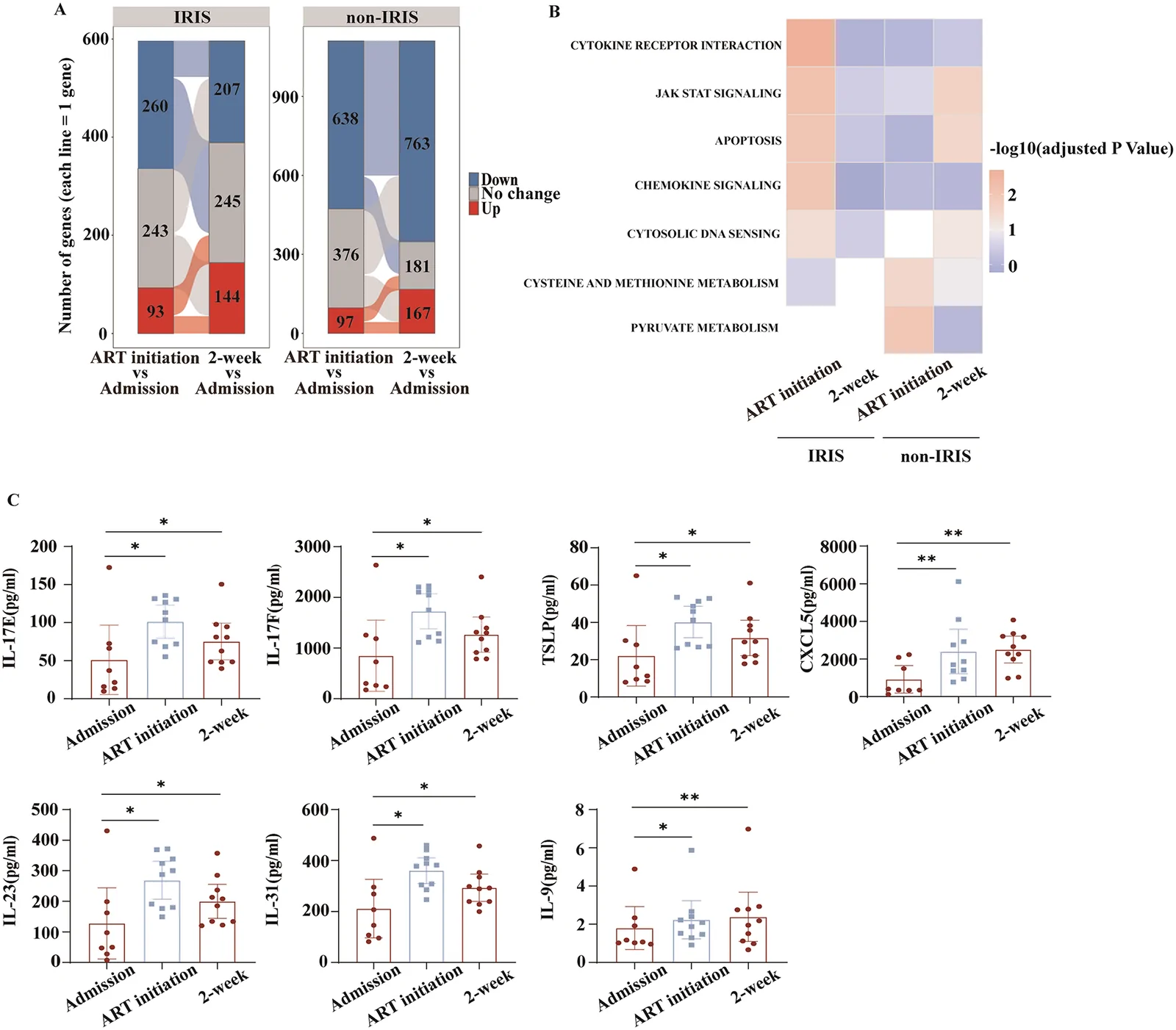
Transcriptomic overlap, pathway enrichment, and cytokine validation. **(A)** A Sankey diagram presents the state transitions of differential expressed genes in the non-IRIS and IRIS groups across three time points: admission, ART initiation, and 2-weeks after ART. Genes are categorized into “Up,” “Down,” and “No change” states based on admission comparisons, with the flow width representing the number of genes transitioning between states. **(B)** KEGG pathway changes across the IRIS and non-IRIS groups at ART initiation and 2 weeks after ART initiation relative to admission. Color bar, -log10(adjusted P Value). **(C)** Cytokine concentrations (IL-17E, IL-17F, TSLP, CXCL5, IL-23, IL-31, and IL-9) in TSM-IRIS at admission, ART initiation, and 2 weeks after initiation. Data are presented as mean ± SD. *P value < 0.05, **P value < 0.01. ART = Antiretroviral therapy; IL = interleukin; TSLP = thymic stromal lymphopoietin; CXCL = C-X-C motif chemokine ligand.

In TSM-IRIS group, using the cutoff criteria of fold-change >2 or <-2 and a *P* value <0.05, 93 upregulated genes and 260 downregulated genes were observed at ART initiation compared to admission, as shown in the alluvial plots in Fig 3A. At 2 weeks after ART initiation, 144 DEGs were upregulated and 207 were downregulated (Fig 3A). The corresponding volcano plots illustrating the gene-level distribution of these DEGs are shown in S6 Fig. Similar trends were observed in non-IRIS group (Fig 3A). To identify pathways that differed between IRIS and non-IRIS patients at different time points, we performed a pathway enrichment analysis of DEGs. Janus kinase–signal transducer and activator of transcription (JAK-STAT) signaling, chemokine signaling, and apoptosis pathways were enriched at ART initiation compared with baseline, but not in non-IRIS group (Fig 3B). This was supported by an increase in interleukin (IL)-17E, IL-17F, Thymic stromal lymphopoietin (TSLP), C-X-C motif chemokine ligand (CXCL)5, IL-23, IL-31, and IL-9 concentrations during the early phase after ART initiation in IRIS group (Fig 3C, S6C-S6E Fig). Collectively, these findings revealed dynamic transcriptional reprogramming and early activation of inflammatory cytokine networks that clearly distinguished patients with TSM-IRIS from non-IRIS patients.

### TSM associated IRIS exhibited early and robust inflammatory activation

**Fig 4:**
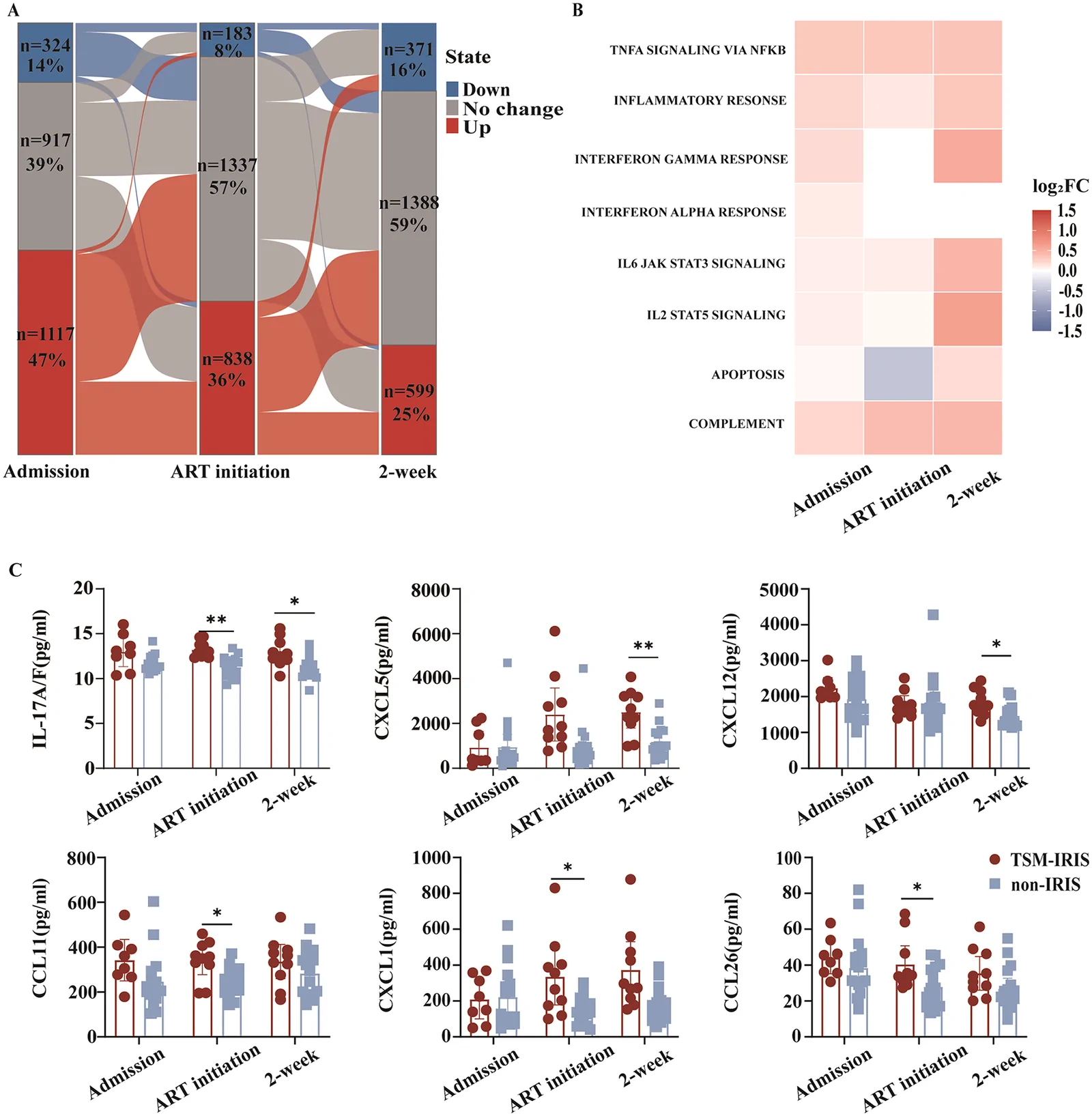
Differential gene expression, pathway enrichment, and cytokine validation between the TSM-IRIS and non-IRIS groups at different time points. **(A)** A Sankey diagram illustrates dynamic changes in differential expressed genes (Up, Down, or No change) between the TSM-IRIS and non-IRIS groups across three time points: admission, ART initiation, and 2 weeks post-initiation. The width of each flow represents the number of genes transitioning between states. **(B)** Heatmap of hallmark pathway activity between the TSM-IRIS and non-IRIS groups at admission, ART initiation, and 2 weeks post-initiation. Color bar, log2FC. **(C)** Comparison of plasma cytokine levels, including IL-17A/F, CXCL5, CXCL12, CCL11, CXCL1, and CCL26, between the TSM-IRIS and non-IRIS groups at admission, ART initiation, and 2 weeks post-initiation. Data are presented as mean ± SD. *P value < 0.05, **P value < 0.01. ART = Antiretroviral therapy; IL = interleukin; CXCL = C-X-C motif chemokine ligand; C-C motif chemokine ligand; C-C motif chemokine = CCL.

The distribution of upregulated, downregulated, and unchanged genes across three time points between two groups were visualized using a Sankey plot (Fig 4A). The IRIS group exhibited a pronounced redistribution of DEGs over time, with a substantial proportion of genes transitioning from an unchanged state at baseline to downregulated states at ART initiation and 2 weeks after ART, indicating transcriptional heterogeneity across different time points in IRIS group compared with non-IRIS group. We then performed gene set enrichment analysis using Hallmark reference datasets to identify pathways that differed between two groups across three time points. Patients with IRIS showed stronger activation of tumor necrotic factor (TNF) α, interferon (IFN) α and γ response, IL6–JAK–STAT3, and IL2–STAT5 signaling pathways at ART initiation and at 2 weeks (Fig 4B). Cytokine quantification demonstrated elevated circulating levels of IL-17A/F, CXCL5, CXCL12, C-C motif chemokine (CCL)11, CXCL1, and CCL26 in patients with IRIS compared with non-IRIS group (Fig 4C and S7-S8 Fig). These cytokines are primarily involved in neutrophil and eosinophil recruitment, and Th17-mediated inflammation, supporting the presence of a robust inflammatory milieu during early immune reconstitution.

Collectively, these findings reveal a distinct pro-inflammatory transcriptomic and cytokine signature associated with TSM-IRIS, providing a molecular framework for its immunopathogenesis.

## Discussion

In our longitudinal study, TSM-IRIS was associated with a distinct pattern of immune reconstitution, characterized by accelerated T cell recovery, altered inhibitory checkpoint expression, and early, sustained inflammatory activation following ART initiation.

Most TSM-IRIS cases manifest within the first 2 weeks of ART, with a markedly earlier onset than the average timing of IRIS onset [11], suggesting that specific host immune characteristics predispose patients to early IRIS onset. This pattern resembles tuberculosis-IRIS, in which early innate immune activation has also been reported [7].

Although our previous study identified higher baseline CD4^+^ T cells in TSM-IRIS [11], baseline CD4^+^ T cells were not significantly different between groups in the present cohort. This discrepancy may be related to the smaller sample size, the requirement for complete longitudinal PBMC and plasma samples, and differences in cohort selection and sampling time points. Similar inconsistencies have been reported in other IRIS entities. Although low baseline CD4^+^ T cells have been associated with an increased risk of TB-IRIS and cryptococcal IRIS [7,12], several prospective immunological studies found no significant baseline CD4^+^ T cell differences between patients who developed IRIS and those who did not [13,14]. Instead, immune recovery dynamics following ART initiation may better reflect susceptibility to TSM-IRIS than baseline CD4 ⁺ T cell level [15]. Additionally, in other IRIS entities, previous studies have suggested that rapid decreases in HIV-1 viral load, differences in ART regimens, and shorter intervals between antifungal therapy and ART initiation may exacerbate immune disequilibrium [16–18]. In our cohort, baseline clinical characteristics, antifungal treatment, ART-related variables, corticosteroid use, and concomitant opportunistic infections were compared between the TSM-IRIS and non-IRIS groups. Therefore, these clinical factors were unlikely to fully account for the immune differences observed in this study. Nevertheless, because of the limited sample size and the clinical complexity of advanced AIDS, residual confounding from treatment-related factors or concomitant infections could not be completely excluded.

On the other hand, differences in immune regulatory pathways may help explain why immune restoration became dysregulated in some patients but remained controlled in others. We found lower PD-1^+^ CD4^+^ T cells at ART initiation. One possibility is that inhibitory pathways that normally restrain immune activation are less effective in patients who develop TSM-IRIS. PD-1 is a key immune checkpoint protein that inhibits T cell function through its expression in CD4^+^ T cells, particularly by suppressing Th1 cytokine production, thereby limiting excessive immune activation [19]. In *T. marneffei* infection, the PD-1 has been implicated in immune evasion and persistent infection, with previous work showing upregulation of this pathway in infected hosts [20]. The lower PD-1 ⁺ CD4 ⁺ T cell frequencies in patients with TSM-IRIS may indicate reduced inhibitory restraint rather than a predominantly exhausted T cells. Because admission and ART initiation represent different pre-ART clinical states, separated by initial antifungal treatment and clinical stabilization, the PD-1 finding may reflect divergent immune trajectories before ART rather than a static baseline difference at hospitalization. These findings suggest that immune regulation may contribute to susceptibility to exaggerated inflammatory responses after ART initiation, although they do not establish a causal role for PD-1 in TSM-IRIS.

Transcriptomic profiling revealed marked temporal heterogeneity, with dynamic shifts in gene expression across sampling time points. Peak levels of several cytokines and chemokines were observed at ART initiation. Therefore, these elevations are unlikely to be mainly caused by ART. Instead, they may reflect an inflammatory state before ART initiation, related to ongoing or recently treated TSM. Their persistence during early ART suggests continuation of this inflammatory state, rather than new induction by ART. These findings suggest that patients with a higher inflammatory state at ART initiation may be more susceptible to developing TSM-IRIS after ART initiation. Consistent with this, pathway enrichment analyses demonstrated the preferential enrichment of inflammatory and immune-related programs, including JAK–STAT signaling, chemokine signaling, and cytokine–receptor interactions, particularly at ART initiation and 2 weeks later. Importantly, these longitudinal findings describe temporal changes after ART initiation, whereas the comparative analyses highlight differences between TSM-IRIS and non-IRIS patients at specific time points. The more prominent inflammatory and chemokine-related signatures observed in the TSM-IRIS group are consistent with an IRIS-associated inflammatory profile rather than a uniform response to ART. This aligns with previous reports linking elevated levels of systemic inflammatory markers before ART with adverse immune outcomes after treatment initiation [21].

Cytokine profiling confirmed these transcriptional signatures at the protein level, with the TSM-IRIS group exhibiting sustained elevation of IL-17 family cytokines, and chemokines such as CXCL5 and CXCL12. Notably, some cytokine differences appeared to be driven by declining levels in the non-IRIS group rather than marked increases in the TSM-IRIS group. This may reflect gradual resolution of inflammation in non-IRIS patients, whereas patients with TSM-IRIS maintained a relatively persistent inflammatory state during early ART. This pattern suggests ongoing inflammatory amplification and continued recruitment of immune effector cells during early ART. These findings indicate that TSM-IRIS is characterized by a temporally coordinated inflammatory response during early immune reconstitution. Similar early inflammatory amplification and chemokine-driven immune cell recruitment have been described in TB-IRIS [22–24], suggesting that dysregulated immune reconstitution can converge on shared inflammatory effector programs across different IRIS entities. In contrast, cryptococcal IRIS often presents later and has been associated with a Th1-skewed rebound and leukocyte recruitment to the central nervous system [25]. These comparisons emphasize that the timing and tissue context of IRIS vary by pathogen, excessive inflammatory immune reconstitution may represent a shared downstream pathway across distinct IRIS.

Our study provides novel insights into the immunological characteristics of TSM-IRIS in patients with AIDS. To our knowledge, this is the first study to systematically integrate cellular, transcriptional, and cytokine analyses in TSM-IRIS. Nevertheless, several limitations should be acknowledged. First, although tuberculosis and cryptococcosis were excluded and other concomitant opportunistic infections were considered during clinical adjudication, residual confounding from concomitant infections could not be completely excluded, particularly in advanced AIDS. Second, steroid exposure may have influenced the transcriptomic findings, although there was no statistically significant difference between groups. Third, the transcriptomic and cytokine analyses were restricted to early post-ART time points, as no IRIS events occurred beyond 4 weeks after ART initiation. Finally, this study included hospitalized patients with HIV-associated TSM, the cohort may represent a more severe and clinically complex subset of TSM-IRIS. Therefore, our analyses focused on describing immunological features during the early phase of immune reconstitution. Although the cohort size was modest owing to the longitudinal nature and clinical rarity of TSM-IRIS, the findings across multiple analytical approaches strengthened our conclusions. Importantly, our study provides a potential immunological framework for understanding susceptibility to TSM-IRIS.

## Conclusions

In conclusion, this study provides immunological characterization of TSM-IRIS in patients with AIDS by integrating analyses of immune cell subsets, transcriptomic signatures, and cytokine profiles, revealing that TSM-IRIS involves dynamic immune alterations accompanied by exaggerated inflammatory responses, and underscoring the potential value of baseline immune profiling and early cytokine dynamics for the candidate immune features for future risk stratification of TSM-IRIS in AIDS patients. Future research should focus on validating these early immune and transcriptional dynamics in larger multicenter cohorts to enable more robust risk stratification. Given the high burden of advanced HIV infection and TSM infection in endemic regions, improved understanding and early recognition of TSM-IRIS may have important public health relevance.

## Supporting information

S1 Fig Representative gating strategy for flow cytometry analysis. Live cells were first selected after exclusion of dead cells using Zombie NIR viability staining. CD3^+^ and CD3^-^ cell populations were then gated. Within CD3^+^ T cells, CD4^+^ and CD8^+^ T-cell subsets were identified. PD-1^+^ CD4^+^ T cells, CD28^+^ CD4^+^ T cells, PD-1^+^ CD8^+^ T cells, and CD28^+^ CD8^+^ T cells were further gated. CD8^+^ T-cell differentiation subsets were defined according to CD45RA and CCR7 expression as naïve CD8^+^ T cells (CD8^+^ CD45RA^+^CCR7^+^), central memory CD8^+^T cells (CD8^+^ CD45RA^-^CCR7^+^), effector memory CD8^+^ T cells (CD8^+^ CD45RA^-^CCR7^-^), and effector CD8^+^ T cells (CD8^+^ CD45RA^+^CCR7^-^). Within CD3^-^ cells, CD11b^+^ and CD11b^-^ populations were gated. Monocyte subsets were identified within CD11b^+^ cells as classical monocytes (CD14^++^CD16^-^), intermediate monocytes (CD14⁺⁺CD16⁺), and non-classical monocytes (CD14^+^ CD16^++^). NK cells and B cells were identified within the CD11b^-^ population as CD16 ⁺ CD56⁺ and CD19 ⁺ cells, respectively.
(TIF)

S2 Fig Comparison of CD4^+^ and CD8^+^ T cell subsets, natural killer (NK), B cells, and monocyte subsets (classical, intermediate, and non-classical) in the TSM-IRIS group at admission, ART initiation, and 2 and 4 weeks after ART initiation. *P *value*  < 0.05.
(TIF)

S3 Fig Comparison of total T cells and T cell subsets in the non-IRIS group at admission, ART initiation, and 2 and 4 weeks after ART initiation. Violin plots depict CD3 ⁺ T cells, CD4 ⁺ T cells, CD8 ⁺ T cells, central memory T cells, effector memory T cells, and naïve T cells. **P value  < 0.01.
(TIF)

S4 Fig Comparison of CD4^+^ and CD8^+^ T cell subsets, NK cells, B cells, and monocyte subsets (classical, intermediate, and non-classical) in the non-IRIS group at admission, ART initiation, 2 weeks and 4 weeks after ART. *P  value < 0.05, **P  value < 0.01.
(TIF)

S5 Fig Comparison of CD4^+^ and CD8^+^ T cell subsets, B cells, NK, and monocyte subsets between the TSM-IRIS and non-IRIS groups at admission, ART initiation, and 2 and 4 weeks after ART initiation. (TIF)

S6 Fig Differential gene expression and cytokine dynamics in the TSM-IRIS group. (A–B) Volcano plots present differential expressed genes in the TSM-IRIS group at ART initiation versus admission (A) and 2 weeks after ART initiation versus admission (B). ns, no significance. signif, significant. (C–E) Longitudinal changes in plasma cytokine levels, including IL-21, IFN-γ1, and CCL24, at admission, ART initiation, and 2 weeks after ART initiation. * *P*  value < 0.05.
(TIF)

S7 Fig Longitudinal changes of cytokine levels in patients with TSM-IRIS. Longitudinal changes of plasma cytokine levels in patients with TSM-IRIS at admission, ART initiation, and 2 weeks after ART initiation, including IL-17A/F (A), CXCL5 (B), CXCL12 (C), CCL11 (D), CXCL1 (E), and CCL26 (F). Each point represents one patient, and connected lines indicate repeated measurements from the same patient.
(TIF)

S8 Fig Longitudinal changes of cytokine levels in patients with non-IRIS. Longitudinal changes in plasma cytokine levels in patients with non-IRIS at admission, ART initiation, and 2 weeks after ART initiation, including IL-17A/F (A), CXCL5 (B), CXCL12 (C), CCL11 (D), CXCL1 (E), and CCL26 (F). Each point represents one patient, and connected lines indicate repeated measurements from the same patient.
(TIF)

S1 Table Longitudinal changes in clinical markers in TSM-IRIS and non-IRIS. (XLSX)

S1 Data Excel spreadsheet containing, in separate sheets, the underlying numerical data and statistical analysis for Figs 1, 2, 3, and 4. (XLSX)
